# The *Phanuromyia galeata* species group (Hymenoptera, Platygastridae, Telenominae): shining a lantern into an unexplored corner of Neotropical diversity

**DOI:** 10.3897/zookeys.663.11554

**Published:** 2017-03-27

**Authors:** Katherine C. Nesheim, Lubomír Masner, Norman F. Johnson

**Affiliations:** 1 Department of Evolution, Ecology, and Organismal Biology, The Ohio State University, 1315 Kinnear Road, Columbus, Ohio, 43212, USA; 2 Agriculture and Agri-Food Canada, K.W. Neatby Building, Ottawa, Ontario K1A 0C6, Canada

**Keywords:** Platygastroidea, parasitoid, species description, key, Neotropical, new species

## Abstract

The *Phanuromyia galeata* species group is delineated and its species richness explored for the first time (Hymenoptera: Platygastridae, Telenominae). Fifteen species are described, all of which are new: *Phanuromyia comata* Nesheim & Masner, **sp. n.** (Brazil), *P. constellata* Nesheim, **sp. n.** (Paraguay), *P. corys* Nesheim & Masner, **sp. n.** (Brazil), *P. cranos* Nesheim & Masner, **sp. n.** (Bolivia, Costa Rica, Ecuador, French Guiana), *P. cudo* Nesheim & Masner, **sp. n.** (Belize, Bolivia, Brazil, Colombia, Costa Rica, Ecuador, French Guiana, Panama, Peru, Trinidad and Tobago, Venezuela), *P. dissidens* Nesheim & Masner, **sp. n.** (Bolivia, Brazil, French Guiana), *P. galeata* Nesheim & Masner, **sp. n.** (Belize, Brazil, Colombia, Costa Rica, Ecuador, El Salvador, French Guiana, Mexico, Peru), *P. galerita* Nesheim & Masner, **sp. n.** (Brazil, Ecuador, French Guiana), *P. hjalmr* Nesheim, **sp. n.** (Bolivia, Costa Rica, Ecuador, Paraguay, Venezuela), *P. krossotos* Nesheim, **sp. n.** (Ecuador), *P. odo* Nesheim, **sp. n.** (Belize, Bolivia, Brazil, Colombia, Costa Rica, Ecuador, El Salvador, French Guiana, Guatemala, Mexico, Panama, Peru, Trinidad and Tobago, Venezuela), *P. pauper* Nesheim, **sp. n.** (Ecuador, Peru), *P. princeps* Nesheim, **sp. n.** (Brazil, Ecuador, French Guiana), *P. tonsura* Nesheim, **sp. n.** (Brazil, Colombia, Ecuador, Paraguay, Peru), *P. tubulifer* Nesheim & Masner, **sp. n.** (Brazil, Guyana).

## Introduction

The subfamily Telenominae (Hymenoptera: Platygastroidea, Platygastridae) is a large group of egg-parasitoid wasps, comprising 905 known species found throughout the world. Traditionally it has been thought to be composed of two major genera, *Telenomus* Haliday and *Trissolcus* Ashmead, and a number of small, morphologically distinctive satellite genera. This view of telenomine diversity was largely based on over 180 years of work on the Holarctic fauna, beginning with Haliday (1833). This scheme, however, has struggled to cope with the diversity of species from the world's tropics.

The genus *Phanuromyia* Dodd was originally described in 1914 on the basis of a single species from southern coastal Queensland, distinguishing it on the basis of the presence of an extruded ovipositor. Dodd later (1916) added a second species from New South Wales. In the years that followed the concept of the genus largely fell into obscurity, being cited only five times in the taxonomic literature for the rest of the century, largely because Dodd's laconic description did little to distinguish the taxon from the many other species in the subfamily and because the original type material in Australia was not studied. Johnson and Musetti (2003) sought to better define the genus, using new characters and a perspective informed by decades of new collections from around the world. Mineo (2006) rejected the recognition of *Phanuromyia*, basing this on the *a priori* assertion that the characters used were only appropriate for distinguishing species groups and not genera. Taekul et al. (2014), however, confirmed that *Phanuromyia* is distinct from *Telenomus* and, further, expanded the concept to embrace species before placed in the *crassiclava* group of *Telenomus* (following Johnson 1984). The data and analyses (Taekul et al. 2014) suggest that *Phanuromyia* is the sister group of *Telenomus*+*Trissolcus* (along with several satellite genera), and the limited host data that are available all indicate that *Phanuromyia* are egg parasitoids of lanternflies and planthoppers in the families Fulgoridae and Flatidae (Hemiptera: Auchenorrhyncha).

In the Neotropics *Phanuromyia* is often the most common telenomine genus to be encountered, even surpassing the abundance of *Telenomus* s.str. However, very few of the species have been formally described. One subset of this diversity is a group of large, elongate, and distinctive species initially recognized by LM as a discrete entity in the fauna of Central and South America: the *galeata* group. The goals of this paper are to document the diversity of the *Phanuromyia galeata* group. The contributions of the authors are as follows: K.C. Nesheim: character definition, species group concept development, species concept development, imaging, key development, manuscript preparation; L. Masner: species group concept development, species concept development, key development; N.F. Johnson: species concept development, manuscript preparation, database design and maintenance.

## Materials and methods

This work is based upon specimens deposited in the following collections, with abbreviations used in the text: CNCI, Canadian National Collection of Insects, Ottawa, Canada; OSUC, C.A. Triplehorn Insect Collection, Columbus, OH. Morphological terminology follows Mikó et al. (2007) and the Hymenoptera Anatomy Ontology (http://portal.hymao.org/projects/32/public/ontology), which is searchable for all morphological terms in this manuscript.

**Information management.** Holotypes are unambiguously identifiable by means of the unique identifier or the red holotype label. The numbers prefixed with “OSUC ” are unique identifiers for the individual specimens. These unique identifiers are associated with their specimens' data in The Ohio State University's Hymenoptera Online database, which can be accessed at http://hol.osu.edu. Searching this database using a specimen's unique identifier will produce all data associated with the specimen. All new species have been prospectively registered with Zoobank as well as the Hymenoptera Name Server (http:// hns.osu.edu).

**Tools.** Images were created using AutoMontage and Combine ZP extended focus software. All images are archived within The Ohio State University's image database (http://specimage.osu.edu). Species descriptions were generated using a database application, vSysLab (http://vsyslab.osu.edu). This application facilitates the construction of taxon character data matrices, the integration of matrices with our existing taxonomic database, and the exportation of data in a variety of file types which can be used in other programs.

**Species concept.** We define species as populations with the potential to interbreed (Mayr 1942). Interbreeding populations will develop a gradient of character states within each morphological character, while distinct separations between character states will exist in non-interbreeding populations (Wild 2004); therefore, species delimitations are made by identifying discrete character states within characters that are present across multiple specimens.

## Results

### Phanuromyia

Taxon classificationAnimaliaHymenopteraPlatygastridae

Dodd

http://zoobank.org/FDEC083E-2450-477E-B678-82F53B317E22

http://bioguid.osu.edu/xbiod_concepts/600

Phanuromyia Dodd, 1914: 121. Original description. Type: Phanuromyia rufobasalis Dodd, by monotypy and original designation. Kieffer 1926: 16, 131 (description, keyed); Muesebeck and Walkley 1956: 384 (citation of type species); Masner, 1976: 79 (taxonomic status); Johnson 1991: 211 (description); Johnson 1992: 564 (catalog, catalog of world species); Johnson and Musetti 2003: 139 (description, synonymy, list of included species); Taekul et al. 2014: 30 (diagnosis, phylogenetic relationships within Telenominae); Veenakumari and Mohanraj 2014: 135, 146 (key to species of India, distribution).
Issidotelenomus Pélov, 1975: 89. Original description. Type: Issidotelenomus obscuripes Pélov, by original designation. Kozlov and Kononova 1983: 137 (junior synonym of Telenomus Haliday); Johnson and Musetti 2003: 140 (junior synonym of Phanuromyia Dodd).

#### Diagnosis.

The three genera *Phanuromyia*, *Telenomus* and *Trissolcus* Ashmead cannot be distinguished on the basis of any single, easily recognized morphological character. Rather, they are recognized by the preponderance of evidence from several characters: presence or absence of sculpture on the medial portion of the frons, length of setation between the ommatidia of the compound eyes, shape of the head, number of clavomeres in the female antenna, presence or absence of notauli on the mesoscutum, presence or absence of sculpture on the disk of the mesoscutellum, form of the sternaulus, shape of the first and second metasomal tergites, and sculpture of the second metasomal tergite. To distinguish *Phanuromyia*, focus should first be placed on the sternaulus. In the large majority of species this is expressed as a line of pits, beginning anteriorly on the mesepisternum near the dorsal apex of the acetabular carina and extending dorsally and posteriorly toward the mesopleural pit. In *Telenomus* and *Trissolcus* the sternaulus may have a single irregularly shaped pit, and its course is otherwise represented by fold or crease in the cuticle. Small individuals of *Phanuromyia*, however, also may have merely a poorly defined line of impression. Supplemental characters to distinguish *Phanuromyia* are eye setation very short or seemingly absent; frontal depression weakly expressed so that the head appears semiglobose in shape; frontal sculpture highly variable, ranging from smooth to sculptured throughout; female antenna with five clavomeres (defined morphologically, see Johnson 1984); notauli absent; mesoscutellar disk sculpture highly variable; T1 strongly transverse; T2 longer than wide; T2 often with distinctive coriaceous to reticulate microsculpture extending beyond the pits marking the position of the antecostal suture and the longitudinal striae arising between those pits. As Dodd (1914) noted, the ovipositor is often exserted a great distance and is easily seen, but this feature is relevant for only a minority of species.

Within *Phanuromyia*, we separate the *galeata* group purely as a practical grouping, and at this point we do not assert its monophyly. The group may be distinguished, first and foremost, by their unusually large body size: most specimens are greater than 2 mm in length. Beyond that, the body is distinctly elongate, T1 in the female is produced into a horn to house the ovipositor, T2 is strongly elongate, as often are the following tergites. The group is strictly Neotropical in distribution, extending from the Isthmus of Tehuantepec in the north to Misiones in southern Paraguay.

#### Key to assist recognition of *Phanuromyia*

**Table:** 

1	Notauli present	***Trissolcus, Telenomus***
–	Notauli absent	**2**
2	Sternaulus indicated by a distinct oblique line of foveolae on the mesepisternum	most ***Phanuromyia***
–	Sternaulus indicated by a shallow fold or by 1 to 2 irregular pits near pro-mesothoracic suture	**3**
3(2)	T2 reticulate beyond basal costae	***Phanuromyia***
–	T2 smooth beyond basal costae	**4**
4(3)	Antennal scrobe absent or weakly impressed, head in lateral profile semiglobose	***Phanuromyia***
–	Antennal scrobe and lateral profile variable	***Trissolcus, Telenomus***

### Phanuromyia comata

Taxon classificationAnimaliaHymenopteraPlatygastridae

Nesheim & Masner sp. n.

http://zoobank.org/01F7E2C9-511E-4ABF-9826-B206A5D2B20E

http://bioguid.osu.edu/xbiod_concepts/403725

Figures 1–6

#### Description.

Female body length: 2.36 mm (n=1).

Median keel on frons: absent. Sculpture of lower frons: with >6 transverse rugae medially. Shape of mandible: slender. Median tooth of mandible: diminished. Frons below median ocellus: with 2 rows of setiferous punctures.

Sculpture on posterior half of mesoscutum: coriaceous to rugulose, at most with fine irregular longitudinal sculpture. Sculpture of anterior half of mesoscutellum: rugose-punctate. Thin median foliaceous lamella on propodeum: absent.

Color of coxae: bright yellow, concolorous with legs.

T1: flat, at most slightly swollen. Anterior margin of T2: with costae or foveolae throughout its width. T2 sculpture: with neither transverse series of small punctures nor scrobiculate lateral areas. Sculpture of T1: entirely costate. Posterior margin of T2: distinctly concave. Number of visible terga past T2: 2 or 3. Setation on T2: consisting of thick patches of lateral setae; consisting of widespread scattered pilosity.

**Figures 1–6.**
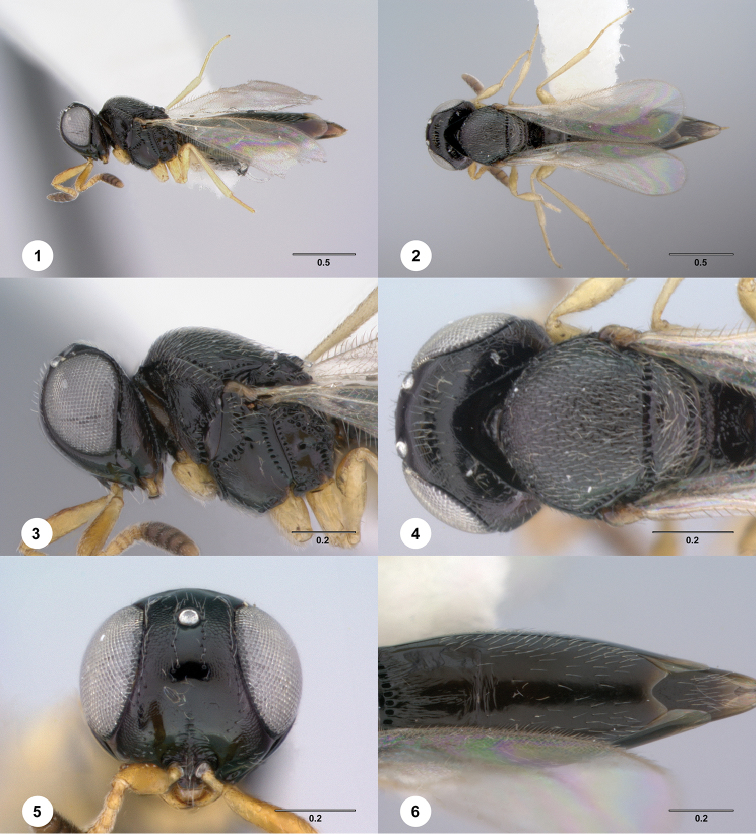
*Phanuromyia comata* ♀ (OSUC 149413), **1** Lateral habitus **2** Dorsal habitus **3** Head, mesosoma, lateral view **4** Head, mesosoma, dorsal view **5** Head, mouthparts, anteroventral view **6** T2–T4, dorsal view. Scale bar in millimeters.

#### Diagnosis.

*Phanuromyia comata* can be recognized by T2 setation consisting of thick patches of lateral setae combined with widespread scattered pilosity dorsally.

#### Etymology.

The name *comata* is derived from the Latin word for having long hair because this species has diagnostic patches of setae. This name is to be used as a participle.

#### Link to distribution map.

[http://hol.osu.edu/map-full.html?id=403725]

#### Material examined.

Holotype, female: **BRAZIL**: MT, 500m, 12°46'S, 55°30'W, Vila Vera, X-1973, M. Alvarenga, OSUC 149413 (deposited in CNCI).

#### Comments.

*Phanuromyia comata* is the only species in the group with widespread pilosity across the entirety of T2, so this character can be used to identify a specimen very quickly.

### Phanuromyia constellata

Taxon classificationAnimaliaHymenopteraPlatygastridae

Nesheim sp. n.

http://zoobank.org/DA85EF19-E1F9-4E63-8164-6437A76CF006

http://bioguid.osu.edu/xbiod_concepts/403720

Figures 7–12

#### Description.

Female body length: 1.43–1.54 mm (n=3).

Median keel on frons: absent. Sculpture of lower frons: with 3–6 transverse rugae medially. Shape of mandible: slender. Median tooth of mandible: diminished. Frons below median ocellus: with two rows of setiferous punctures converging medially and then diverging ventrally.

Sculpture on posterior half of mesoscutum: coriaceous to rugulose, at most with fine irregular longitudinal sculpture. Sculpture of anterior half of mesoscutellum: smooth. Thin median foliaceous lamella on propodeum: absent.

Color of coxae: bright yellow, concolorous with legs.

T1: swollen in posterior half. Anterior margin of T2: with costae or foveolae throughout its width. T2 sculpture: with neither transverse series of small punctures nor scrobiculate lateral areas. Sculpture of T1: entirely costate. Posterior margin of T2: straight; only slightly concave. Number of visible terga past T2: 4 or 5. Setation on T2: limited to at most 1 row of setae posteriorly and sparse setation laterally.

**Figures 7–12.**
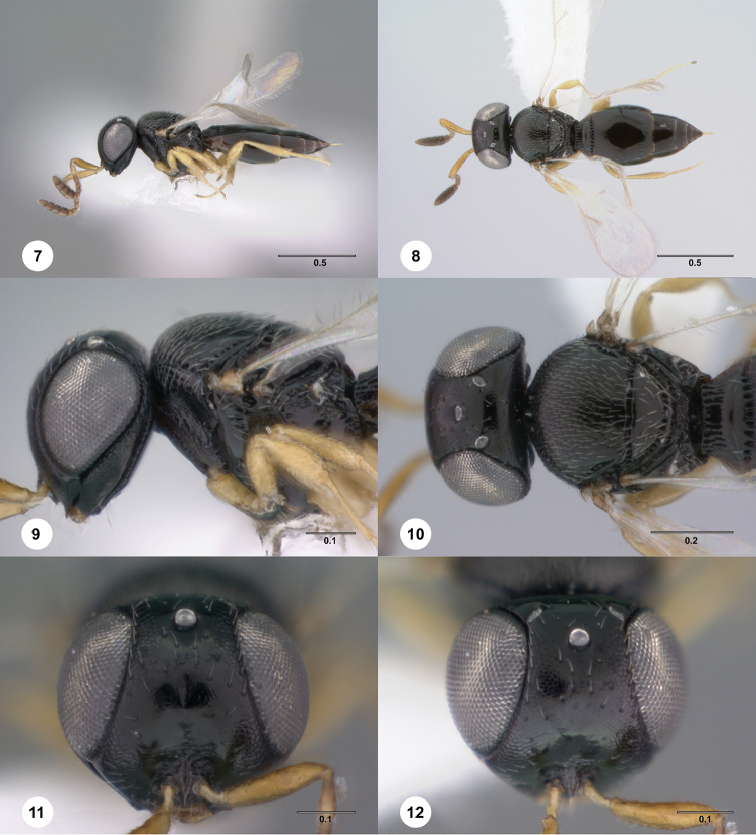
*Phanuromyia constellata* ♀ (OSUC 322906), **7** Lateral habitus **8** Dorsal habitus **9** Head, mesosoma, lateral view **10** Head, mesosoma, dorsal view **11** Head, mouthparts, anteroventral view **12** Head, ventral view. Scale bar in millimeters.

#### Diagnosis.

*Phanuromyia constellate* can be recognized by the swirling pattern of setiferous punctures on the frons.

#### Etymology.

The name *constellata* is derived from the Latin word for constellation because the pattern of punctures on this species’ frons is reminiscent of stars in the sky. This name is to be used as a noun in apposition.

#### Link to distribution map.

[http://hol.osu.edu/map-full.html?id=403720]

#### Material examined.

Holotype, female: **PARAGUAY**: Canindeyú Dept., Jejuí-mí, wet grazing floor, MT1, Bosque Mbaracayú Natural Reserve, 29.V–11.VI.1996, Malaise trap, A. C. F. Costa, OSUC 322906 (deposited in OSUC). *Paratypes*: **PARAGUAY**: 2 females, OSUC 322905, 322907 (OSUC).

### Phanuromyia corys

Taxon classificationAnimaliaHymenopteraPlatygastridae

Nesheim & Masner sp. n.

http://zoobank.org/777F2663-8058-4618-8062-B2E0C0E30161

http://bioguid.osu.edu/xbiod_concepts/389325

Figures 13–18

#### Description.

Female body length: 2.80–3.05 mm (n=3).

Median keel on frons: absent. Sculpture of lower frons: with multiple transverse rugae. Shape of mandible: slender. Median tooth of mandible: diminished. Frons below median ocellus: with two rows of setiferous punctures converging ventrally.

**Figures 13–18.**
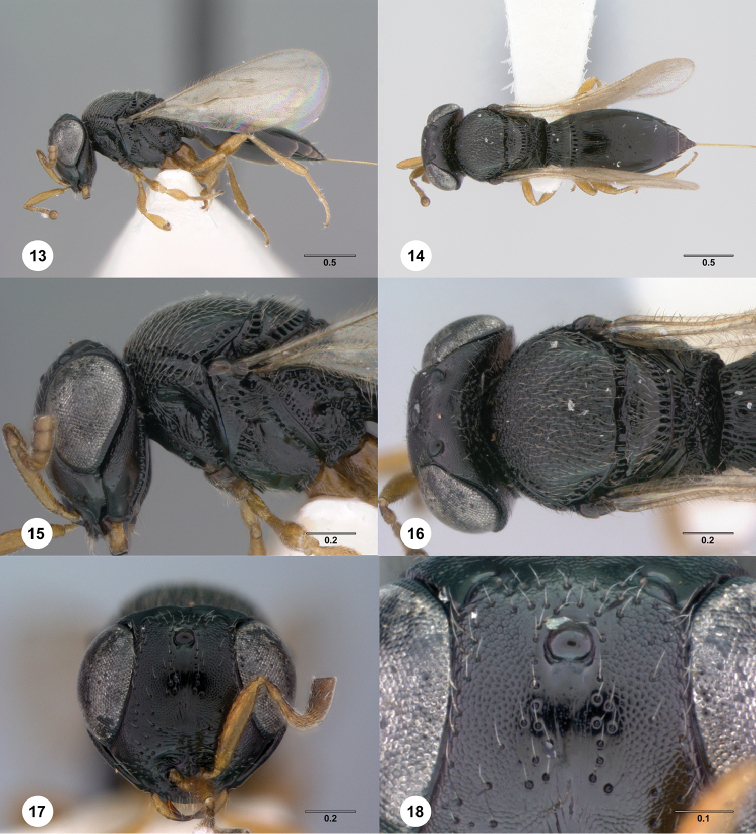
*Phanuromyia corys* ♀ (OSUC 149359), **13** Lateral habitus **14** Dorsal habitus **15** Head, mesosoma, lateral view **16** Head, mesosoma, dorsal view **17** Head, mouthparts, anteroventral view **18** Frons, anteroventral view. Scale bar in millimeters.

Sculpture on posterior half of mesoscutum: coriaceous to rugulose, at most with fine irregular longitudinal sculpture. Sculpture of anterior half of mesoscutellum: rugose-punctate. Thin median foliaceous lamella on propodeum: absent.

Color of coxae: bright yellow, concolorous with legs.

T1: flat, at most slightly swollen. Anterior margin of T2: with costae or foveolae throughout its width. T2 sculpture: with neither transverse series of small punctures nor scrobiculate lateral areas. Sculpture of T1: entirely costate. Posterior margin of T2: straight; only slightly concave. Number of visible terga past T2: 4 or 5. Setation on T2: limited to at most 1 row of setae posteriorly and sparse setation laterally.

#### Diagnosis.

*Phanuromyia corys* can be recognized by the two rows of setiferous punctures converging ventrally on the frons.

#### Etymology.

The name *corys* is derived from a Greek word for helmet because this species has a large head evoking the image of a knight wearing a helmet. This name is to be used as a noun in apposition.

#### Link to distribution map.

[http://hol.osu.edu/map-full.html?id=389325]

#### Material examined.

Holotype, female: **BRAZIL**: RJ, Silva Jardim, VIII-1974, F. M. Oliveira, OSUC 550201 (deposited in CNCI). *Paratypes*: **BRAZIL**: 3 females, OSUC 149358–149360 (CNCI).

#### Comments.

This species is recognizable by its large size, only the largest specimens of *P. odo* reach over 2.7 mm in length. *Phanuromyia corys* may be distinguished from *P. odo* by the converging lines of setiferous punctures on the frons and the straight, transverse apical margin of T2.

### Phanuromyia cranos

Taxon classificationAnimaliaHymenopteraPlatygastridae

Nesheim & Masner sp. n.

http://zoobank.org/59383F22-EFB5-4638-B7BC-7BBAEB35D567

http://bioguid.osu.edu/xbiod_concepts/389326

Figures 19–24

#### Description.

Female body length: 2.21–2.50 mm (n=20).

Median keel on frons: absent. Sculpture of lower frons: with multiple transverse rugae; with irregular rugosity. Shape of mandible: slender. Median tooth of mandible: as large as adjacent teeth. Frons below median ocellus: with 2 perfectly parallel rows of setiferous punctures.

**Figures 19–24.**
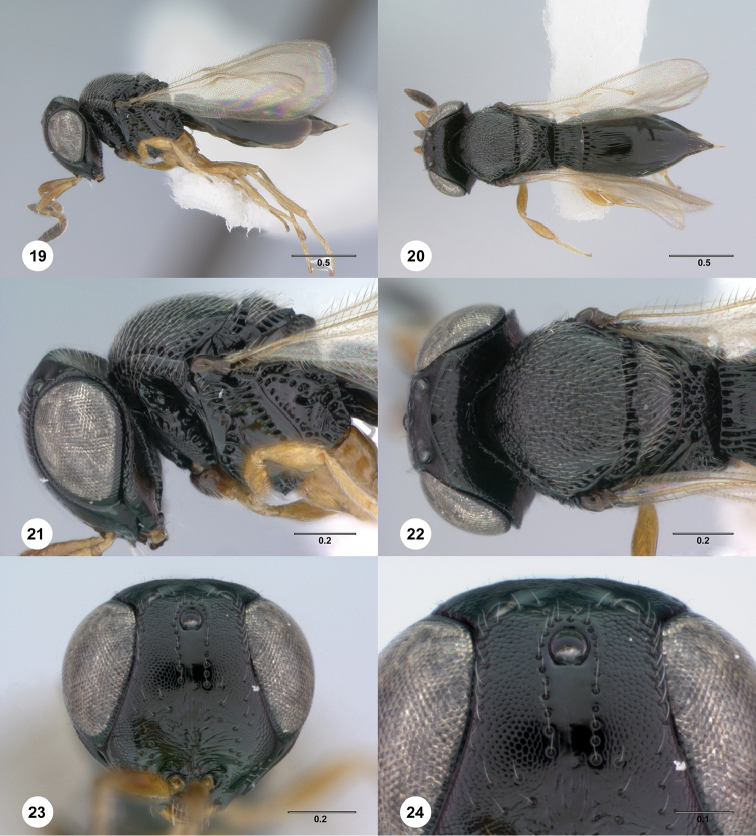
*Phanuromyia cranos* ♀ (OSUC 550034), **19** Lateral habitus **20** Dorsal habitus **21** Head, mesosoma, lateral view **22** Head, mesosoma, dorsal view **23** Head, mouthparts, anteroventral view **24** Frons, anteroventral view. Scale bar in millimeters.

Sculpture on posterior half of mesoscutum: coriaceous to rugulose, at most with fine irregular longitudinal sculpture. Sculpture of anterior half of mesoscutellum: rugose-punctate. Thin median foliaceous lamella on propodeum: absent.

Color of coxae: bright yellow, concolorous with legs.

T1: flat, at most slightly swollen. Anterior margin of T2: with costae or foveolae throughout its width. T2 sculpture: with neither transverse series of small punctures nor scrobiculate lateral areas. Sculpture of T1: entirely costate. Posterior margin of T2: straight; only slightly concave. Number of visible terga past T2: 2 or 3. Setation on T2: limited to at most 1 row of setae posteriorly and sparse setation laterally.

#### Diagnosis.

*Phanuromyia cranos* can be recognized by the 2 perfectly parallel rows of setiferous punctures on the frons.

#### Etymology.

The name *cranos* is derived from a Greek word for helmet because this species has a large head evoking the image of a knight wearing a helmet. This name is to be used as a noun in apposition.

#### Link to distribution map.

[http://hol.osu.edu/map-full.html?id=389326]

#### Material examined.

Holotype, female: **ECUADOR**: Sucumbíos Prov., Napo River, 270m, 00°30'S, 76°30'W, Sacha Lodge, 3.IV–13.IV.1994, Malaise trap, P. Hibbs, OSUC 550028 (deposited in CNCI). *Paratypes*: (21 females) **BOLIVIA**: 4 females, OSUC 149423, 550038–550040 (CNCI). **COSTA RICA**: 5 females, OSUC 149421, 164007, 550031–550032, 550035 (CNCI). **ECUADOR**: 11 females, OSUC 149420, 149422, 164006, 320967, 550027, 550029–550030, 550033–550034, 550036–550037 (CNCI). **FRENCH GUIANA**: 1 female, OSUC 550111 (CNCI).

#### Comments.

*Phanuromyia cranos* can be identified most quickly by the distinctive pattern of setiferous punctures on its frons. The setiferous frontal puncture of *P. odo* may at times appear similar, but *P. odo* has the apical margin of T2 distinctly concave.

### Phanuromyia cudo

Taxon classificationAnimaliaHymenopteraPlatygastridae

Nesheim & Masner sp. n.

http://zoobank.org/9785C5ED-ABDC-405E-B771-999C038B87CC

http://bioguid.osu.edu/xbiod_concepts/389327

Figures 25–30

#### Description.

Female body length: 1.33–1.82 mm (n=20).

Median keel on frons: absent. Sculpture of lower frons: with 3–6 transverse rugae medially. Shape of mandible: slender. Median tooth of mandible: diminished. Frons below median ocellus: with 2 rows of setiferous punctures.

**Figures 25–30.**
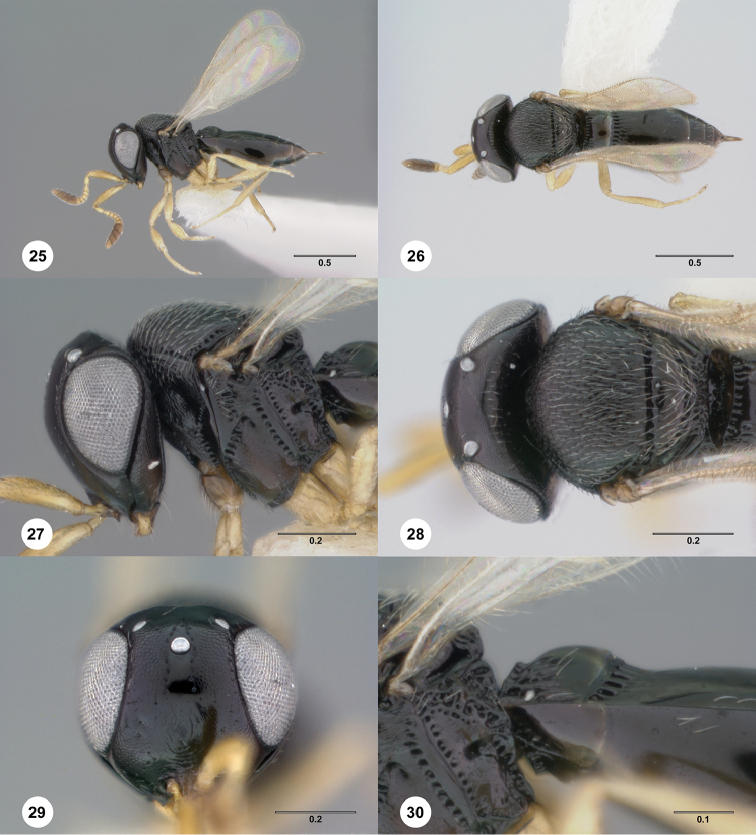
*Phanuromyia cudo* ♀ (OSUC 550006), **25** Lateral habitus **26** Dorsal habitus **27** Head, mesosoma, lateral view **28** Head, mesosoma, dorsal view **29** Head, anteroventral view **30** T1–T2, lateral view. Scale bar in millimeters.

Sculpture on posterior half of mesoscutum: coriaceous to rugulose, at most with fine irregular longitudinal sculpture. Sculpture of anterior half of mesoscutellum: smooth. Thin median foliaceous lamella on propodeum: absent.

Color of coxae: bright yellow, concolorous with legs.

T1: distinctly swollen throughout entire length. Anterior margin of T2: with costae or foveolae throughout its width. T2 sculpture: with neither transverse series of small punctures nor scrobiculate lateral areas. Sculpture of T1: almost entirely smooth. Posterior margin of T2: straight. Number of visible terga past T2: 3 or 4. Setation on T2: limited to at most 1 row of setae posteriorly and sparse setation laterally.

#### Diagnosis.

*Phanuromyia cudo* can be recognized by the swelling of the entire T1 segment.

#### Etymology.

The name *cudo* is derived from the Latin word for a helmet made of raw skin because this species has a large head evoking the image of a knight wearing a helmet. This name is to be used as a noun in apposition.

#### Link to distribution map.

[http://hol.osu.edu/map-full.html?id=389327]

#### Material examined.

Holotype, female: **COSTA RICA**: Heredia Prov., La Selva Biological Station, 100m, X-1992, Malaise trap, P. Hanson, OSUC 549938 (deposited in CNCI). *Paratypes*: (129 females) **BELIZE**: 1 female, OSUC 550084 (CNCI). **BOLIVIA**: 10 females, OSUC 149379, 149405–149406, 550016–550019, 550080–550082 (CNCI). **BRAZIL**: 2 females, OSUC 149400, 550012 (CNCI). **COLOMBIA**: 1 female, OSUC 149407 (CNCI). **COSTA RICA**: 37 females, OSUC 149381–149382, 149388, 149390–149391, 149394–149395, 149399, 149408, 549929–549935, 549937, 549956–549957, 549964–549965, 549978–549979, 549983, 549987, 550007, 550010, 550014, 550020–550026, 550079, 575268 (CNCI). **ECUADOR**: 65 females, OSUC 149387, 149393, 149397, 240600, 549936, 549939–549946, 549948–549955, 549958–549963, 549966–549970, 549973–549977, 549981–549982, 549984–549986, 549988–549996, 549998–550006, 550008, 550013, 550113–550115 (CNCI). **FRENCH GUIANA**: 1 female, OSUC 550099 (CNCI). **PANAMA**: 7 females, OSUC 149380, 149384, 149389, 149401, 149409, 549971, 549997 (CNCI). **PERU**: 2 females, OSUC 549980, 550107 (CNCI). **TRINIDAD AND TOBAGO**: 2 females, OSUC 149383, 550015 (CNCI). **VENEZUELA**: 1 female, OSUC 149403 (CNCI).

#### Comments.

This species exhibits variation in several characters, but can be reliably diagnosed by the swollen appearance of the entirety of T2.

### Phanuromyia dissidens

Taxon classificationAnimaliaHymenopteraPlatygastridae

Nesheim & Masner sp. n.

http://zoobank.org/6F72B99A-A5AA-4F40-AEA0-4197D000A302

http://bioguid.osu.edu/xbiod_concepts/403721

Figures 30 , 31–36

#### Description.

Female body length: 1.07–2.22 mm (n=4).

Median keel on frons: present. Sculpture of lower frons: with multiple transverse rugae. Shape of mandible: slender. Median tooth of mandible: diminished. Frons below median ocellus: evenly covered with setiferous punctures.

**Figures 31–36.**
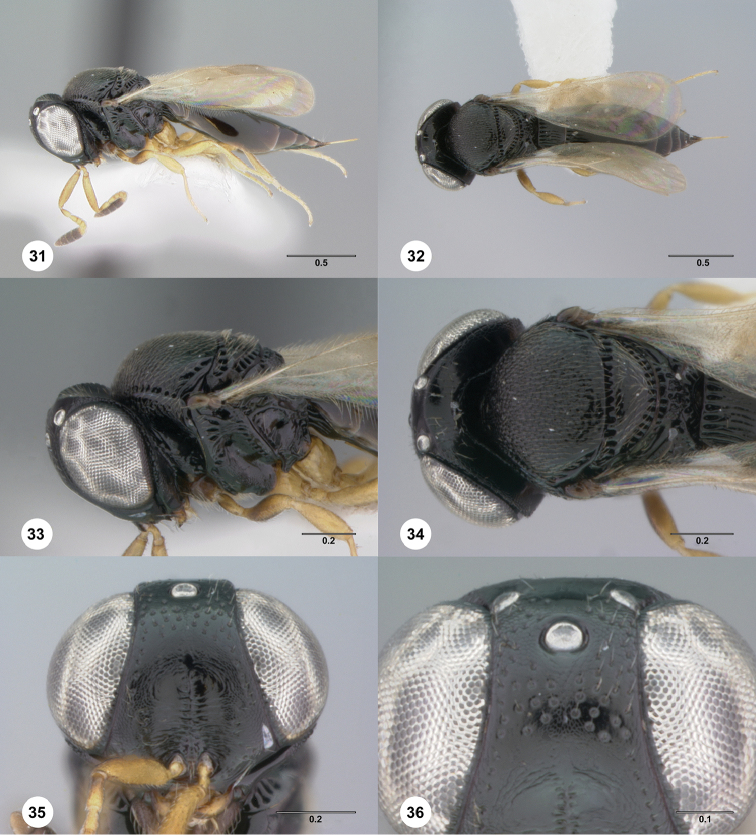
*Phanuromyia dissidens* ♀ (OSUC 149412), **31** Lateral habitus **32** Dorsal habitus **33** Head, mesosoma, lateral view **34** Head, mesosoma, dorsal view **35** Head, mouthparts, anteroventral view **36** Frons, anteroventral view. Scale bar in millimeters.

Sculpture on posterior half of mesoscutum: coriaceous to rugulose, at most with fine irregular longitudinal sculpture. Sculpture of anterior half of mesoscutellum: rugose-punctate. Thin median foliaceous lamella on propodeum: absent.

Color of coxae: bright yellow, concolorous with legs.

T1: flat, at most slightly swollen. Anterior margin of T2: with costae or foveolae throughout its width. T2 sculpture: with neither transverse series of small punctures nor scrobiculate lateral areas. Sculpture of T1: costate at sides, smooth medially; entirely costate. Posterior margin of T2: straight; slightly convex. Number of visible terga past T2: 5. Setation on T2: limited to at most 1 row of setae posteriorly and sparse setation laterally.

#### Diagnosis.

*Phanuromyia dissidens* can be recognized by the even covering of setiferous punctures on the frons.

#### Etymology.

The name *dissidens* is derived from the Latin word for differing because this species has an evenly punctured frons, differentiating it from the other species in the group. This name is to be used as a participle.

#### Link to distribution map.

[http://hol.osu.edu/map-full.html?id=403721]

#### Material examined.

Holotype, female: **BRAZIL**: MT, 500m, 12°46'S, 55°30'W, Vila Vera, X-1973, M. Alvarenga, OSUC 149412 (deposited in CNCI). *Paratypes*: (3 females) **BOLIVIA**: 1 female, OSUC 550077 (CNCI). **FRENCH GUIANA**: 2 females, OSUC 550105, 550110 (CNCI).

#### Comments.

This species most closely resembles *P. krossotos*. *Phanuromyia dissedens* may be distinguished by the absence of patches of setae on laterally on T2.

### Phanuromyia galeata

Taxon classificationAnimaliaHymenopteraPlatygastridae

Nesheim & Masner sp. n.

http://zoobank.org/F0693029-5388-4AE5-A406-067BD87D5179

http://bioguid.osu.edu/xbiod_concepts/386058

Figures 37–42

#### Description.

Female body length: 2.02–2.44 mm (n=15). Male body length: 1.84–2.10 mm (n=5).

Median keel on frons: present. Sculpture of lower frons: with multiple transverse rugae. Shape of mandible: slender. Median tooth of mandible: diminished. Frons below median ocellus: without setiferous punctures.

**Figures 37–42.**
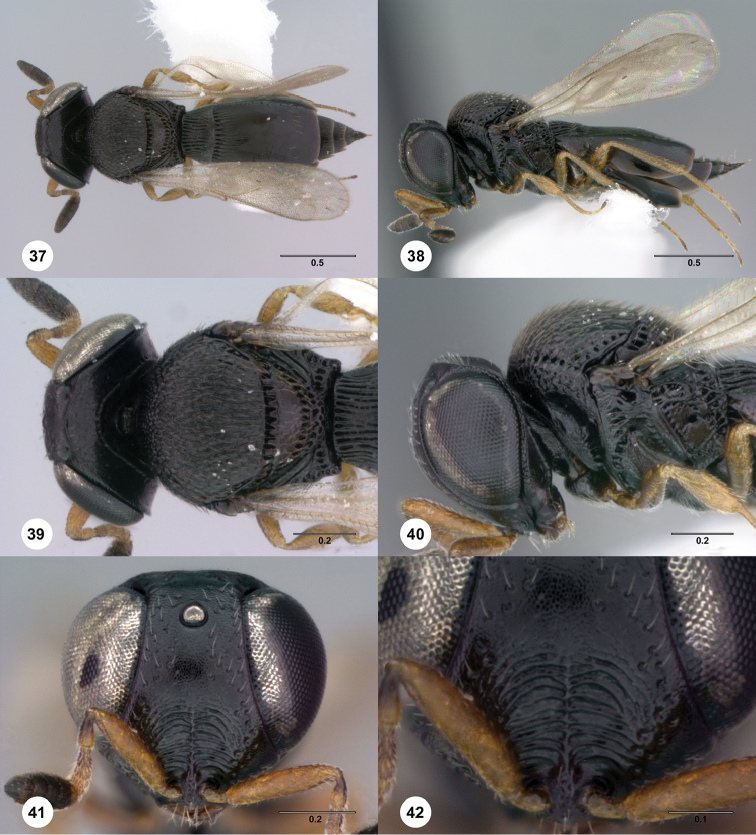
*Phanuromyia galeata* ♀ (OSUC 555798), **37** Dorsal habitus **38** Lateral habitus **39** Head, mesosoma, dorsal view **40** Head, mesosoma, lateral view **41** Head, mouthparts, anteroventral view **42** Frons, anteroventral view. Scale bar in millimeters.

Sculpture on posterior half of mesoscutum: with strong, parallel longitudinal keels. Sculpture of anterior half of mesoscutellum: smooth. Thin median foliaceous lamella on propodeum: absent.

Color of coxae: dark brown to black, contrasting with legs.

T1: flat, at most slightly swollen. Anterior margin of T2: with costae or foveolae throughout its width. T2 sculpture: with neither transverse series of small punctures nor scrobiculate lateral areas. Sculpture of T1: entirely costate. Posterior margin of T2: straight; slightly convex. Number of visible terga past T2: 5. Setation on T2: limited to at most 1 row of setae posteriorly and sparse setation laterally.

#### Diagnosis.

*Phanuromyia galeata* can be recognized by the median keel on the frons.

#### Etymology.

The name *galeata* is derived from a Latin word for helmet because this species has a large head evoking the image of a knight wearing a helmet. This name is to be used as a noun in apposition.

#### Link to distribution map.

[http://hol.osu.edu/map-full.html?id=386058]

#### Material examined.

Holotype, female: **COSTA RICA**: Puntarenas Prov., road to Rincón, 24 km W Pan-American Highway, 200 m, III-1989 – V-1989, Hanson & Gauld, OSUC 550198 (deposited in CNCI). *Paratypes*: (62 females, 5 males) **BELIZE**: 1 female, OSUC 550083 (CNCI). **BRAZIL**: 3 females, OSUC 149315–149316, 550188 (CNCI). **COLOMBIA**: 2 females, OSUC 149320 (CNCI); OSUC 170507 (OSUC). **COSTA RICA**: 21 females, 3 males, OSUC 149313, 149319, 149321–149324, 149326, 359303, 550087, 550093–550097, 550189–550197, 550199 (CNCI). **ECUADOR**: 18 females, 2 males, OSUC 149310–149311, 149325, 550170–550184, 550187, 550200 (CNCI). **EL SALVADOR**: 5 females, OSUC 550088–550092 (CNCI). **FRENCH GUIANA**: 6 females, OSUC 149317–149318, 550102, 550116, 555798, 555801 (CNCI). **MEXICO**: 1 female, OSUC 320968 (CNCI). **PERU**: 5 females, OSUC 149312, 149314, 550106, 550185–550186 (CNCI).

#### Comments.

This species most closely resembles *P. galerita*, but the two can be easily distinguished from each other by comparing the mandibles: *P. galeata* has a slender mandible with a small median tooth, while *P. galerita* has much broader mandibles and a median tooth as large as the outer teeth.

### Phanuromyia galerita

Taxon classificationAnimaliaHymenopteraPlatygastridae

Nesheim & Masner sp. n.

http://zoobank.org/9EE257B5-1447-4290-BB70-9C6026337A3D

http://bioguid.osu.edu/xbiod_concepts/389328

Figures 43–48

#### Description.

Female body length: 2.31–2.55 mm (n=5).

Median keel on frons: absent. Sculpture of lower frons: with irregular rugosity. Shape of mandible: broad. Median tooth of mandible: as large as adjacent teeth. Frons below median ocellus: without setiferous punctures.

**Figures 43–48.**
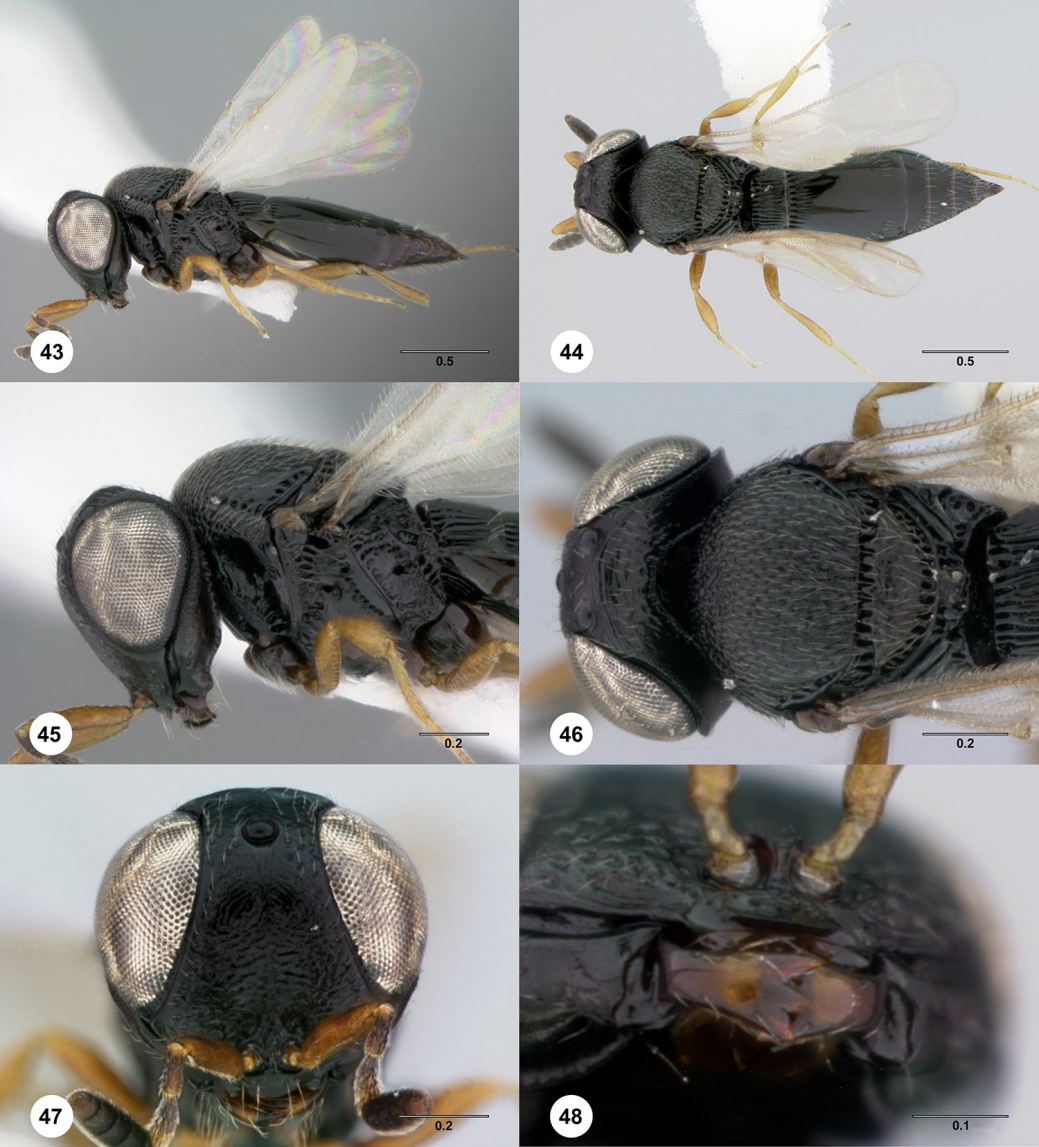
*Phanuromyia galerita* ♀ (OSUC 550202), **43** Lateral habitus **44** Dorsal habitus **45** Head, mesosoma, lateral view **46** Head, mesosoma, dorsal view **47** Head, mouthparts, anteroventral view **48** Mouthparts, ventral view. Scale bar in millimeters.

Sculpture on posterior half of mesoscutum: with strong, parallel longitudinal keels. Sculpture of anterior half of mesoscutellum: rugose-punctate. Thin median foliaceous lamella on propodeum: absent.

Color of coxae: dark brown to black, contrasting with legs.

T1: flat, at most slightly swollen. Anterior margin of T2: with costae or foveolae throughout its width. T2 sculpture: with neither transverse series of small punctures nor scrobiculate lateral areas. Sculpture of T1: entirely costate. Posterior margin of T2: straight; slightly convex. Number of visible terga past T2: 5. Setation on T2: limited to at most 1 row of setae posteriorly and sparse setation laterally.

#### Diagnosis.

*Phanuromyia galerita* can be recognized by the median tooth of the mandible, which is as large as the adjacent teeth.

#### Etymology.

The name *galerita* is derived from the Latin word for wearing a hood because this species has a large head evoking the image of a hooded figure. This name is to be used as a noun in apposition. This name is to be used as a participle.

#### Link to distribution map.

[http://hol.osu.edu/map-full.html?id=389328]

#### Material examined.

Holotype, female: **ECUADOR**: Sucumbíos Prov., 270m, 00°30'S, 76°30'W, Sacha Lodge, 13.VI–23.VI.1994, Malaise trap, P. Hibbs, OSUC 149327 (deposited in CNCI). *Paratypes*: (4 females) **BRAZIL**: 1 female, OSUC 149328 (CNCI). **ECUADOR**: 2 females, OSUC 550202–550203 (CNCI). **FRENCH GUIANA**: 1 female, OSUC 550101 (CNCI).

#### Comments.

This species most closely resembles *P. galeata*, but the two can be easily distinguished from each other by comparing the mandibles: *P. galeata* has a slender mandible with a small median tooth, while *P. galerita* has much broader mandibles and a median tooth as large as the outer teeth.

### Phanuromyia hjalmr

Taxon classificationAnimaliaHymenopteraPlatygastridae

Nesheim sp. n.

http://zoobank.org/CDCE8232-4502-4479-9317-5A7DE79295A7

http://bioguid.osu.edu/xbiod_concepts/403730

Figures 49–54

#### Description.

Female body length: 1.19–1.99 mm (n=6).

Median keel on frons: absent. Sculpture of lower frons: with irregular rugosity; with 3–6 transverse rugae medially. Shape of mandible: slender. Median tooth of mandible: diminished. Frons below median ocellus: with 2 rows of setiferous punctures.

**Figures 49–54.**
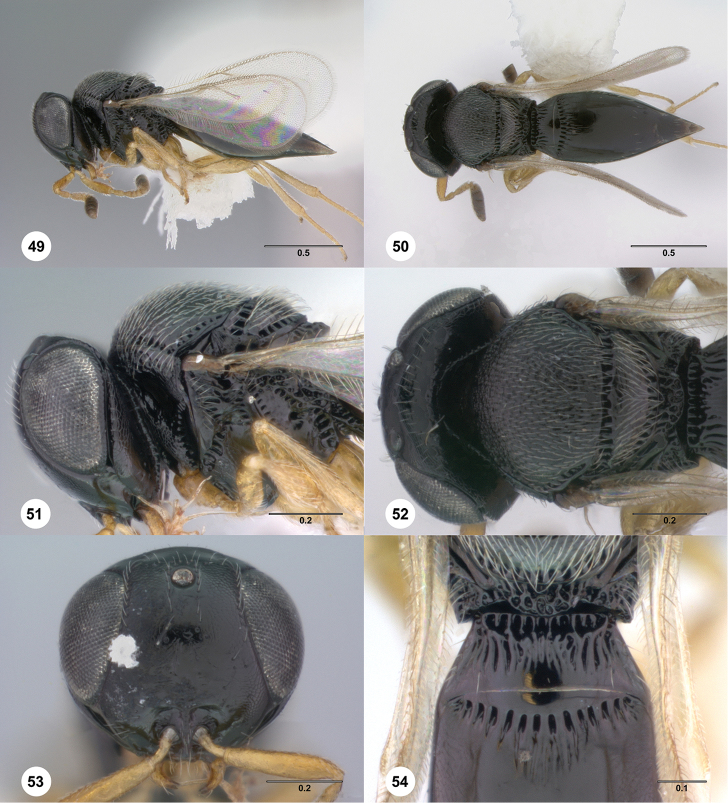
*Phanuromyia hjalmr* ♀ (OSUC 550078), **49** Lateral habitus **50** Dorsal habitus **51** Head, mesosoma, lateral view **52** Head, mesosoma, dorsal view **53** Head, mouthparts, anteroventral view **54** T1–T2, dorsal view. Scale bar in millimeters.

Sculpture on posterior half of mesoscutum: coriaceous to rugulose, at most with fine irregular longitudinal sculpture. Sculpture of anterior half of mesoscutellum: rugose-punctate. Thin median foliaceous lamella on propodeum: absent.

Color of coxae: bright yellow, concolorous with legs.

T1: flat, at most slightly swollen. Anterior margin of T2: with costae or foveolae throughout its width. T2 sculpture: with neither transverse series of small punctures nor scrobiculate lateral areas. Sculpture of T1: evenly costate across anterior 1/3 to 1/2, smooth in remaining apical portion. Posterior margin of T2: distinctly concave. Number of visible terga past T2: 2 or 3. Setation on T2: limited to at most 1 row of setae posteriorly and sparse setation laterally.

#### Diagnosis.

*Phanuromyia hjalmr* can be recognized by the sculpture of T1, which is evenly costate across the anterior 1/3 to 1/2 and smooth in remaining apical portion, combined with the distinctly concave posterior margin of T2.

#### Etymology.

The name *hjalmr* is derived from the Old Norse word for helmet because this species has a large head evoking the image of a knight wearing a helmet. This name is to be used as a noun in apposition.

#### Link to distribution map.

[http://hol.osu.edu/map-full.html?id=403730]

#### Material examined.

Holotype, female: **BOLIVIA**: La Paz Dept., Nor Yungas Prov., Coroico, cloud forest, B-03, El Bagante, 1500m, 18.IV.1997, screen sweeping, L. Masner, OSUC 149417 (deposited in CNCI). *Paratypes*: (5 females) **COSTA RICA**: 1 female, OSUC 550078 (CNCI). **ECUADOR**: 1 female, OSUC 550050 (CNCI). **PARAGUAY**: 2 females, OSUC 322901, 324322 (OSUC). **VENEZUELA**: 1 female, OSUC 149392 (CNCI).

#### Comments.

This species most closely resembles *P. tonsura*, but the two species can be distinguished by their T1 sculpture: *P. tonsura* has the medial portion of T1 smooth from its anterior to posterior margin, while *P. hjalmr* is sculptured across the entire anterior third of T1.

### Phanuromyia krossotos

Taxon classificationAnimaliaHymenopteraPlatygastridae

Nesheim sp. n.

http://zoobank.org/99ACD36B-BD3C-4B7D-BF87-4780C014CFCD

http://bioguid.osu.edu/xbiod_concepts/389330

Figures 55–60

#### Description.

Male body length: 1.46 mm (n=1).

Median keel on frons: absent. Sculpture of lower frons: without rugosity. Shape of mandible: slender. Median tooth of mandible: diminished. Frons below median ocellus: with setiferous punctures only medially.

**Figures 55–60.**
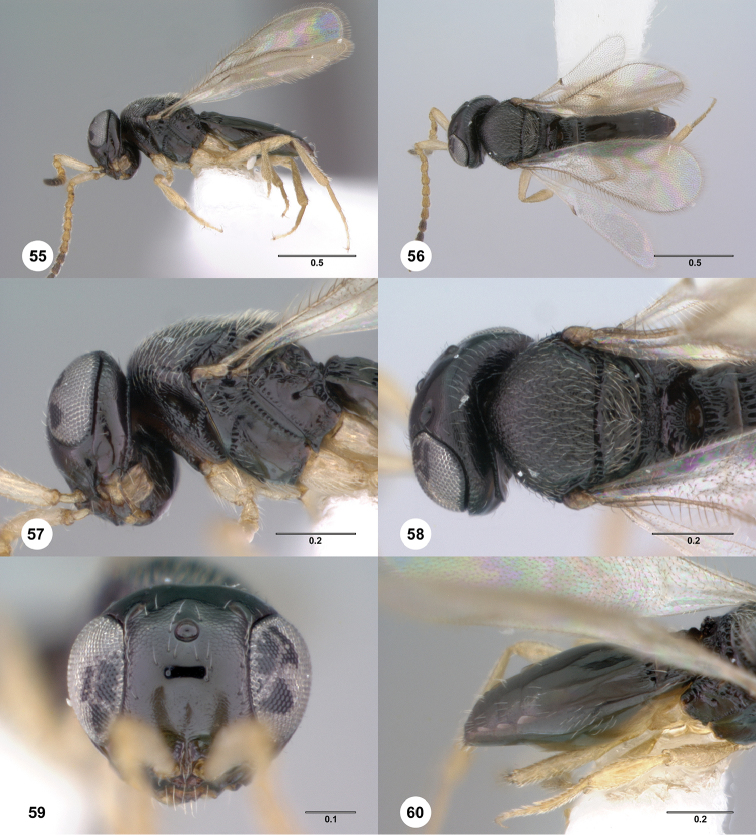
*Phanuromyia krossotos* ♂ (OSUC 550046), **55** Lateral habitus **56** Dorsal habitus **57** Head, mesosoma, lateral view **58** Head, mesosoma, dorsal view **59** Head, mouthparts, anteroventral view **60** Metasoma, lateral view. Scale bar in millimeters.

Sculpture on posterior half of mesoscutum: coriaceous to rugulose, at most with fine irregular longitudinal sculpture. Sculpture of anterior half of mesoscutellum: rugose-punctate. Thin median foliaceous lamella on propodeum: absent.

Color of coxae: bright yellow, concolorous with legs.

T1: flat, at most slightly swollen. Anterior margin of T2: with costae or foveolae throughout its width. T2 sculpture: with neither transverse series of small punctures nor scrobiculate lateral areas. Sculpture of T1: evenly costate across anterior 1/3 to 1/2, smooth in remaining apical portion. Posterior margin of T2: only slightly concave. Number of visible terga past T2: 5. Setation on T2: consisting of thick patches of lateral setae.

#### Diagnosis.

*Phanuromyia krossotos* can be recognized by the thick lateral patches of setae on T2.

#### Etymology.

The name *krossotos* is derived from the Greek word for fringed because this species has a distinctive fringe of lateral setae on T2. This name is to be used as an adjective.

#### Link to distribution map.

[http://hol.osu.edu/map-full.html?id=389330]

#### Material examined.

Holotype, male: **ECUADOR**: Sucumbíos Prov., Napo River, 290m, 00°05'S, 76°05'W, Sacha Lodge, 14.III–24.III.1994, Malaise trap, P. Hibbs, OSUC 550046 (deposited in CNCI).

#### Comments.

This species most closely resembles *P. dissidens*. This species most closely resembles *P. dissidens*. *Phanuromyia krossotos* may be distinguished by the presence of patches of setae on laterally on T2.

### Phanuromyia odo

Taxon classificationAnimaliaHymenopteraPlatygastridae

Nesheim sp. n.

http://zoobank.org/561818C9-9A51-492A-84C5-910AF4BDDA62

http://bioguid.osu.edu/xbiod_concepts/389324

Figures 61–66

#### Description.

Female body length: 1.51–2.78 mm (n=22).

Median keel on frons: absent. Sculpture of lower frons: with 3–6 transverse rugae medially; with irregular rugosity medially. Shape of mandible: slender. Median tooth of mandible: diminished. Frons below median ocellus: with 2 rows of setiferous punctures.

**Figures 61–66.**
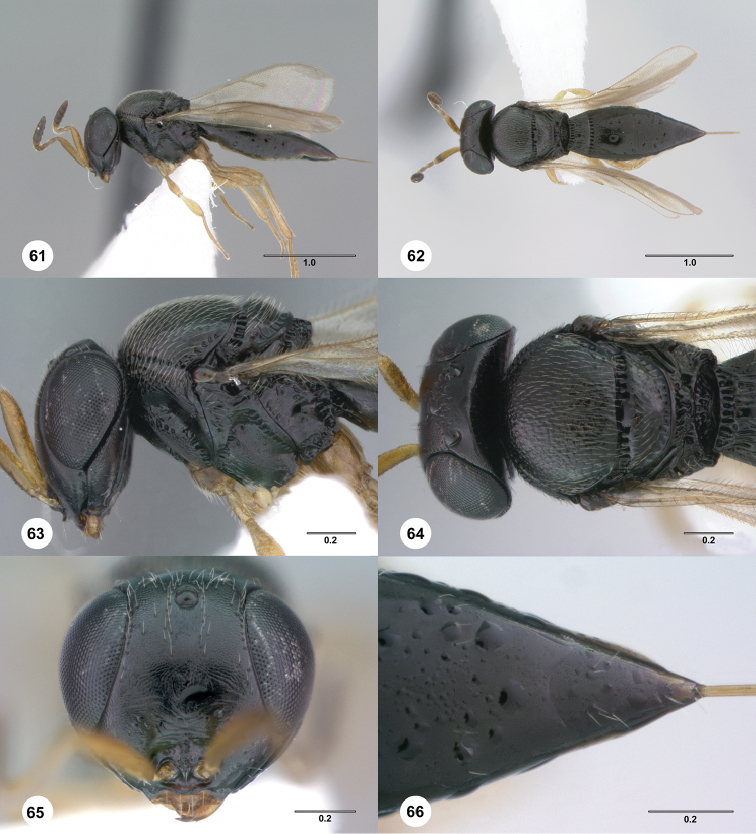
*Phanuromyia odo* ♀ (OSUC 550248), **61** Lateral habitus **62** Dorsal habitus **63** Head, mesosoma, lateral view **64** Head, mesosoma, dorsal view **65** Head, mouthparts, anteroventral view **66** T2–T6, dorsal view. Scale bar in millimeters.

Sculpture on posterior half of mesoscutum: coriaceous to rugulose, at most with fine irregular longitudinal sculpture. Sculpture of anterior half of mesoscutellum: rugose-punctate. Thin median foliaceous lamella on propodeum: absent.

Color of coxae: bright yellow, concolorous with legs.

T1: flat, at most slightly swollen. Anterior margin of T2: with costae or foveolae throughout its width. T2 sculpture: with neither transverse series of small punctures nor scrobiculate lateral areas. Sculpture of T1: entirely costate. Posterior margin of T2: distinctly concave. Number of visible terga past T2: 2 or 3. Setation on T2: limited to at most 1 row of setae posteriorly and sparse setation laterally.

#### Diagnosis.

*Phanuromyia odo* can be recognized by the distinctly concave posterior margin of T2 combined with the entirely costate sculpture of T1.

#### Etymology.

The name *odo* is derived from the name of the Changeling in the popular television series *Star Trek: Deep Space Nine* because this species has variable morphology. This name is to be used as a noun in apposition.

#### Link to distribution map.

[http://hol.osu.edu/map-full.html?id=389324]

#### Material examined.

Holotype, female: **COSTA RICA**: Heredia Prov., La Selva Biological Station, 1.V–8.V.1989, Malaise trap, B. V. Brown, OSUC 149335 (deposited in CNCI). *Paratypes*: (175 females, 2 males) **BELIZE**: 2 females, OSUC 149352, 550252 (CNCI). **BOLIVIA**: 6 females, OSUC 149354, 149372, 149375, 550122, 550127–550128 (CNCI). **BRAZIL**: 9 females, OSUC 149341–149342, 149345, 149378, 550075–550076, 550232, 550238 (CNCI); OSUC 151125 (OSUC). **COLOMBIA**: 1 female, OSUC 149373 (CNCI). **COSTA RICA**: 100 females, 1 male, OSUC 149329–149331, 149333–149334, 149336–149337, 149348, 149350, 149353, 149355–149357, 149362–149364, 149367, 149369, 149377, 164001, 550085–550086, 550098, 550123–550126, 550129–550133, 550135, 550148, 550150, 550157–550158, 550204–550219, 550221–550231, 550233–550237, 550239, 550241–550251, 550253–550268, 550270, 575269–575270 (CNCI); OSUC 575271 (OSUC). **ECUADOR**: 43 females, 1 male, OSUC 149338–149340, 149343–149344, 149347, 149351, 149361, 149365–149366, 240601, 320966, 550134, 550136–550147, 550149, 550151–550156, 550159–550160, 550162–550169, 550220, 550240 (CNCI). **EL SALVADOR**: 1 female, OSUC 149374 (CNCI). **FRENCH GUIANA**: 6 females, OSUC 550103–550104, 550108, 555797, 555799–555800 (CNCI). **GUATEMALA**: 1 female, OSUC 149371 (CNCI). **MEXICO**: 1 female, OSUC 149346 (CNCI). **PANAMA**: 1 female, OSUC 149368 (CNCI). **PERU**: 1 female, OSUC 149376 (CNCI). **TRINIDAD AND TOBAGO**: 1 female, OSUC 550161 (CNCI). **VENEZUELA**: 2 females, OSUC 149332, 149349 (CNCI).

#### Comments.

**Figure 67.**
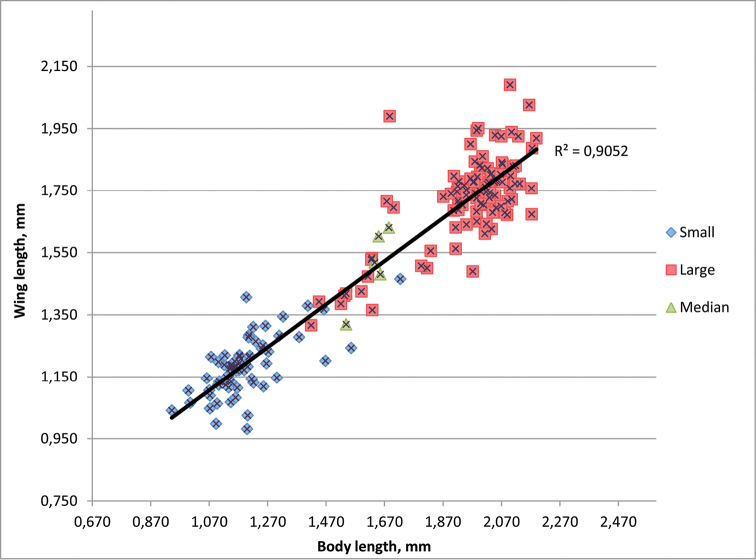
The specimens identified as *Phanuromyia odo* were originally split into two separate species, here labeled “small” and “large.” The trend line is calculated based on data for all specimens combined. The continuity in both variables and the overlap in specimens divided a priori into small and large categories led to the conclusion that there is insufficient evidence to separate these specimens into two species.

The specimens here referred to as *Phanuromyia odo* were originally split into two provisional species. This was based upon differences in the relative length of the wings, specifically, whether the wings reached beyond the apex of the metasoma. Figure 67 illustrates the relationship between body size and wing length: larger specimens indeed have relatively shorter wings. However, there is no distinctive gap in the ratio between the two. Therefore, we treat them here as a single species.

### Phanuromyia pauper

Taxon classificationAnimaliaHymenopteraPlatygastridae

Nesheim & Masner sp. n.

http://zoobank.org/561818C9-9A51-492A-84C5-910AF4BDDA62

http://bioguid.osu.edu/xbiod_concepts/389329

Figures 68–73

#### Description.

Female body length: 1.31–1.62 mm (n=19).

Median keel on frons: absent. Sculpture of lower frons: with irregular rugosity medially. Shape of mandible: slender. Median tooth of mandible: diminished. Frons below median ocellus: with 2 rows of setiferous punctures.

**Figures 68–73.**
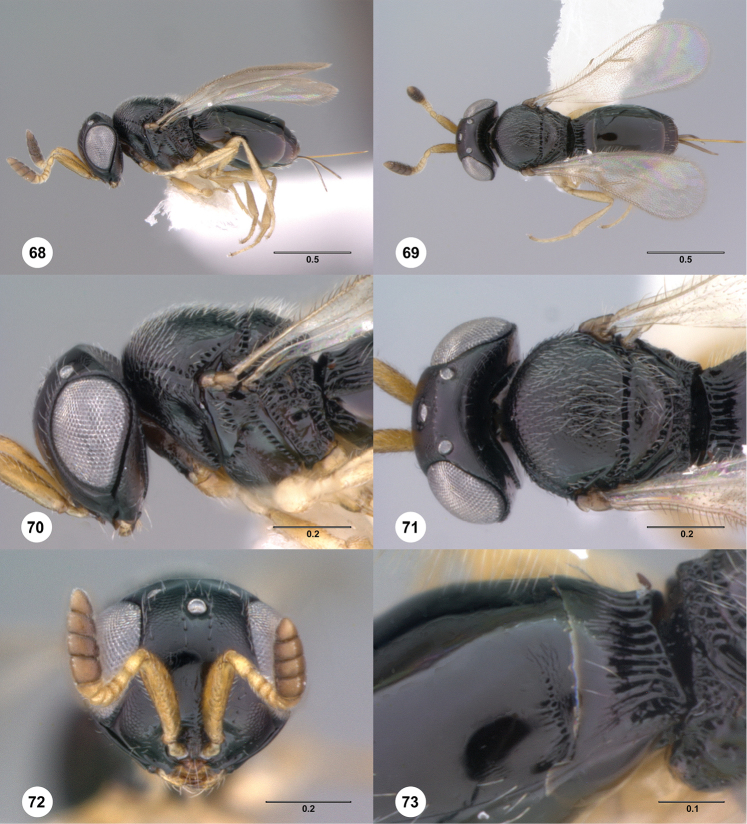
*Phanuromyia pauper* ♀ (OSUC 550066), **68** Lateral habitus **69** Dorsal habitus **70** Head, mesosoma, lateral view **71** Head, mesosoma, dorsal view **72** Head, mouthparts, anteroventral view **73** T1–T2, lateral view. Scale bar in millimeters.

Sculpture on posterior half of mesoscutum: coriaceous to rugulose, at most with fine irregular longitudinal sculpture. Sculpture of anterior half of mesoscutellum: rugose-punctate. Thin median foliaceous lamella on propodeum: absent.

Color of coxae: bright yellow, concolorous with legs.

T1: flat, at most slightly swollen. Anterior margin of T2: medially without costae or foveolae. T2 sculpture: laterally scrobiculate, smooth medially. Sculpture of T1: evenly costate across anterior 1/3 to 1/2, smooth in remaining apical portion. Posterior margin of T2: straight. Number of visible terga past T2: 4 or 5. Setation on T2: limited to at most 1 row of setae posteriorly and sparse setation laterally.

#### Diagnosis.

*Phanuromyia pauper* can be recognized by the T2 sculpture, which is scrobiculate laterally and smooth medially.

#### Etymology.

The name *pauper* refers to the lack of longitudinal costae on the base of T2. This name is to be used as a noun in apposition.

#### Link to distribution map.

[http://hol.osu.edu/map-full.html?id=389329]

#### Material examined.

Holotype, female: **PERU**: Madre de Dios Reg., canopy, 290m, 12°50'S, 69°17'W, Tambopata National Reserve, III-1983 – IX-1983, fogging, T. L. Erwin, OSUC 149427 (deposited in CNCI). *Paratypes*: **ECUADOR**: 18 females, OSUC 149396, 149424–149425, 164002, 549972, 550011, 550062–550067, 550069–550074 (CNCI).

#### Comments.

This species very closely resembles *P. princeps*, although the two species can be distinguished easily by the sculpture of T2: *P. princeps* has a complete scrobiculate angled “belt” while *P. pauper* only has lateral costae.

### Phanuromyia princeps

Taxon classificationAnimaliaHymenopteraPlatygastridae

Nesheim sp. n.

http://zoobank.org/26939117-0EDA-42F8-9930-2108E2DE1686

http://bioguid.osu.edu/xbiod_concepts/403732

Figures 74–79

#### Description.

Female body length: 1.38–1.48 mm (n=10).

Median keel on frons: absent. Sculpture of lower frons: with 3–6 transverse rugae medially. Shape of mandible: slender. Median tooth of mandible: diminished. Frons below median ocellus: with 2 rows of setiferous punctures.

**Figures 74–79.**
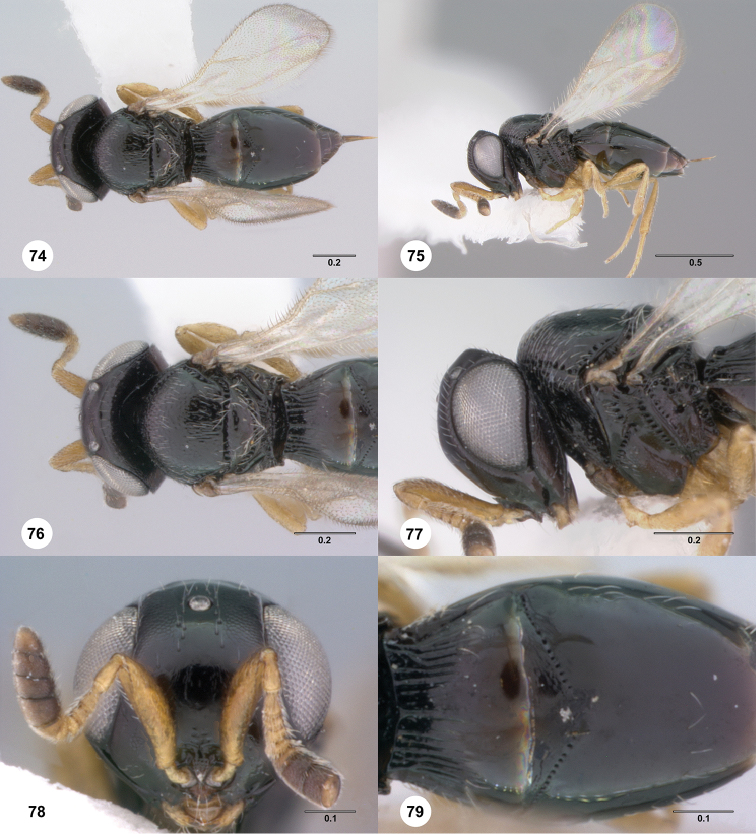
*Phanuromyia princeps* ♀ (OSUC 151126), **74** Dorsal habitus **75** Lateral habitus **76** Head, mesosoma, dorsal view **77** Head, mesosoma, lateral view **78** Head, mouthparts, anteroventral view **79** T1–T3, dorsal view. Scale bar in millimeters.

Sculpture on posterior half of mesoscutum: coriaceous to rugulose, at most with fine irregular longitudinal sculpture. Sculpture of anterior half of mesoscutellum: rugose-punctate. Thin median foliaceous lamella on propodeum: absent.

Color of coxae: bright yellow, concolorous with legs.

T1: flat, at most slightly swollen. Anterior margin of T2: medially without costae or foveolae. T2 sculpture: with transverse series of small punctures in shape of incurved chevron. Sculpture of T1: evenly costate across anterior 1/3 to 1/2, smooth in remaining apical portion. Posterior margin of T2: straight; slightly convex. Number of visible terga past T2: 4 or 5. Setation on T2: limited to at most 1 row of setae posteriorly and sparse setation laterally.

#### Diagnosis.

*Phanuromyia princeps* can be recognized by the T2 sculpture, which consists of a transverse series of small punctures in the shape of an incurved chevron.

#### Etymology.

The name *princeps* is derived from the prince character in the book *The Prince and the Pauper* by Mark Twain in reference to its similarity to *P. pauper*. This name is to be used as a noun in apposition.

#### Link to distribution map.

[http://hol.osu.edu/map-full.html?id=403732]

#### Material examined.

Holotype, female: **BRAZIL**: BA, Sapiranga Reserve, sweeping 13, 12°33'27.3"S 38°03'05"W, Mata de São João, 24.VII.2001, sweeping, M. T. Tavares et al., OSUC 150922 (deposited in OSUC). *Paratypes*: (9 females) **BRAZIL**: 4 females, OSUC 150923, 151077, 151098, 151126 (OSUC). **ECUADOR**: 1 female, OSUC 550068 (CNCI). **FRENCH GUIANA**: 4 females, OSUC 149426, 550100, 550109, 550112 (CNCI).

#### Comments.

This species very closely resembles *P. pauper*, although the two species can be distinguished easily by the sculpture on T2: *P. princeps* has a complete scrobiculate angled “belt,” while *P. pauper* only has lateral costae.

### Phanuromyia tonsura

Taxon classificationAnimaliaHymenopteraPlatygastridae

Nesheim sp. n.

http://zoobank.org/90880ED0-8CEA-4475-855D-D42583686F2E

http://bioguid.osu.edu/xbiod_concepts/403728

Figures 80–85

#### Description.

Female body length: 1.32–1.77 mm (n=20).

Median keel on frons: absent. Sculpture of lower frons: with irregular rugosity medially. Shape of mandible: slender. Median tooth of mandible: diminished. Frons below median ocellus: with 2 rows of setiferous punctures.

**Figures 80–85.**
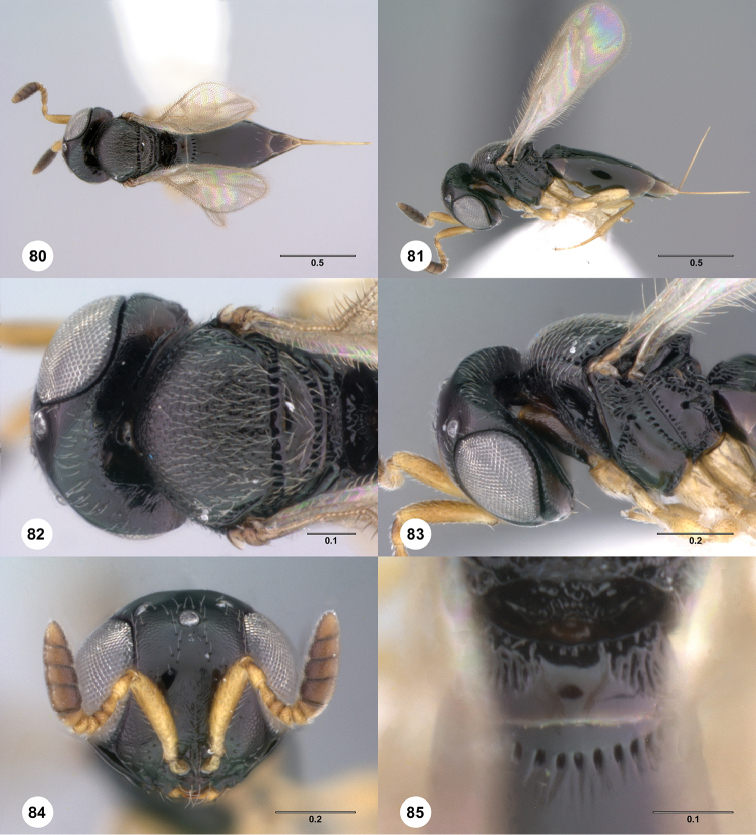
*Phanuromyia tonsura* ♀ (OSUC 149418), **80** Dorsal habitus **81** Lateral habitus **82** Head, mesosoma, dorsal view **83** Head, mesosoma, lateral view **84** Head, mouthparts, anteroventral view **85** T1–T2, dorsal view. Scale bar in millimeters.

Sculpture on posterior half of mesoscutum: coriaceous to rugulose, at most with fine irregular longitudinal sculpture. Sculpture of anterior half of mesoscutellum: uncertain, smooth. Thin median foliaceous lamella on propodeum: absent.

Color of coxae: bright yellow, concolorous with legs.

T1: flat, at most slightly swollen. Anterior margin of T2: with costae or foveolae throughout its width. T2 sculpture: with neither transverse series of small punctures nor scrobiculate lateral areas. Sculpture of T1: costate at sides, smooth medially. Posterior margin of T2: distinctly concave. Number of visible terga past T2: 2 or 3. Setation on T2: limited to at most 1 row of setae posteriorly and sparse setation laterally.

#### Diagnosis.

*Phanuromyia tonsura* can be recognized by the sculpture of T1, which is costate laterally and smooth medially.

#### Etymology.

The name *tonsura* is derived from the Latin word for a shearing and refers to the tonsure hairstyle often worn by monks, because this species has a smooth “bald” area in the middle of T1. This name is to be used as a noun in apposition.

#### Link to distribution map.

[http://hol.osu.edu/map-full.html?id=403728]

#### Material examined.

Holotype, female: **ECUADOR**: Sucumbíos Prov., Napo River, 270m, 00°30'S, 76°30'W, Sacha Lodge, 10.X–21.X.1994, Malaise trap, P. Hibbs, OSUC 550269 (deposited in CNCI). *Paratypes*: (33 females) **BRAZIL**: 1 female, OSUC 149370 (CNCI). **COLOMBIA**: 1 female, OSUC 149404 (CNCI). **ECUADOR**: 26 females, OSUC 149386, 149414–149416, 164005, 240606, 549947, 550041–550045, 550047–550049, 550051–550061 (CNCI). **PARAGUAY**: 3 females, OSUC 322900, 322902, 322904 (OSUC). **PERU**: 2 females, OSUC 149402, 149418 (CNCI).

#### Comments.

This species most closely resembles *P. hjalmr*, but the two species can be easily distinguished by their T1 sculpture: *P. tonsura* has the medial portion of T1 smooth from its anterior to posterior margin, while *P. hjalmr* is sculptured across the entire anterior third of T1.

### Phanuromyia tubulifer

Taxon classificationAnimaliaHymenopteraPlatygastridae

Nesheim & Masner sp. n.

http://zoobank.org/A2D35F8B-369A-466A-9D88-217E0BFE090B

http://bioguid.osu.edu/xbiod_concepts/403723

Figures 86–91

#### Description.

Female body length: 2.24–2.26 mm (n=2).

Median keel on frons: absent. Sculpture of lower frons: with multiple transverse rugae. Shape of mandible: slender. Median tooth of mandible: diminished. Frons below median ocellus: with 2 rows of setiferous punctures.

**Figures 86–91.**
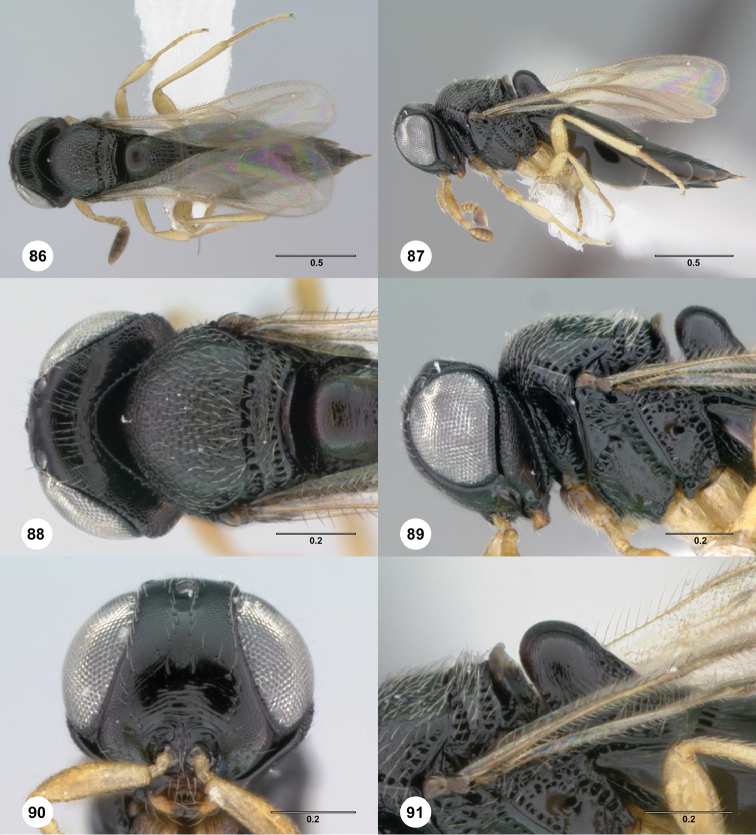
*Phanuromyia tubulifer* ♀ (OSUC 149410), **86** Dorsal habitus **87** Lateral habitus **88** Head, mesosoma, dorsal view **89** Head, mesosoma, lateral view **90** Head, mouthparts, anteroventral view **91** Mesosoma, T1, lateral view. Scale bar in millimeters.

Sculpture on posterior half of mesoscutum: coriaceous to rugulose, at most with fine irregular longitudinal sculpture. Sculpture of anterior half of mesoscutellum: uncertain, rugose-punctate. Thin median foliaceous lamella on propodeum: present.

Color of coxae: bright yellow, concolorous with legs.

T1: with distinct, tubular horn reaching higher than metascutellum. Anterior margin of T2: with costae or foveolae throughout its width. T2 sculpture: with neither transverse series of small punctures nor scrobiculate lateral areas. Sculpture of T1: entirely costate. Posterior margin of T2: straight; only slightly concave. Number of visible terga past T2: 3 or 4. Setation on T2: limited to at most 1 row of setae posteriorly and sparse setation laterally.

#### Diagnosis.

*Phanuromyia tubulifer* can be recognized by the distinct, tubular horn on T1 and the thin foliaceous lamella present medially on the propodeum.

#### Etymology.

The name *tubulifer* refers to the presence of the diagnostic tubular horn on T1 in this species. This name is to be used as a noun in apposition.

#### Link to distribution map.

[http://hol.osu.edu/map-full.html?id=403723]

#### Material examined.

Holotype, female: **GUYANA**: Potaro-Siparuni Reg., 100–200 m, 04°40'19"N, 58°41'04"W, Iwokrama Forest Reserve, V-2001 – VI-2001, flight intercept trap, R. Brooks & Z. Falin, OSUC 149410 (deposited in CNCI). *Paratype*: **BRAZIL**: 1 female, OSUC 149411 (CNCI).

#### Comments.

This species is distinct due to its tubular horn, although it is unknown whether the males express this character to any significant degree.

### Key to species of the *Phanuromyia galeata* group

**Table:** 

1	Posterior half to 2/3 of mesoscutum with strong parallel, longitudinal keels (best viewed from behind at 45-degree angle) (Figs 39, 46); all coxae dark brown to black, contrasting with the otherwise bright yellow legs (Fig. 38)	**2**
–	Posterior half of mesoscutum at most with fine, irregular longitudinal sculpture, usually coriaceous to rugulose (Fig. 34); all coxae bright yellow, concolorous with remaining segments of legs (Fig. 1)	**3**
2(1)	Median keel present on frons; lower frons with multiple transverse rugae; disc of mesoscutellum smooth; mandible slender with median tooth smaller than anterior or posterior tooth (Figs 37–42) (Belize, Brazil, Colombia, Costa Rica, Ecuador, El Salvador, French Guiana, Mexico, Peru)	***P. galeata* sp. n.** ♂♀
–	No median keel on frons; lower frons irregularly rugose; mesoscutellum rugose punctate at least in anterior half; mandible broad with middle tooth as large as adjacent teeth (Figs 43–48) (Brazil, Ecuador, French Guiana)	***P. galerita* sp. n.** ♀
3(1)	Frons below median ocellus with an irregular pattern of setiferous punctures (Figs 35, 59)	**4**
–	Frons below median ocellus with 2 parallel or subparallel rows of setiferous punctures (Fig. 90)	**5**
4(3)	Frons below median ocellus evenly covered with setiferous punctures; no lateral patch of setae on T2; body length 1.0 to 2.3 mm (Figs 31–36) (French Guiana, Bolivia, Brazil)	***P. dissidens* sp. n.** ♀♂
–	Frons below median ocellus with setiferous punctures only medially; lateral patch of 15–20 setae present on T2; body length 1.5 mm (Figs 55–60) (Ecuador)	***P. krossotos* sp. n.** ♂
5(4)	Female T1 with distinct tubular horn; propodeum with foliaceous lamella anterior to T1 horn (Figs 86–91) (Brazil, Guyana)	***P. tubulifer* sp. n.** ♀
–	Female T1 at most moderately swollen (Fig. 38); propodeum without median transverse lamella (Figs 25, 38, 81)	**6**
6(5)	T2 with a line of foveae that begins at the anterolateral margin of the sclerite and extends obliquely toward the midline (Fig. 74)	**7**
–	Anterior margin of T2 longitudinally costate or with fovea that extend transversely toward the midline (Fig. 37)	**8**
7(6)	T2 with series of small punctures in the shape of an incurved chevron (Figs 74–79) (Brazil, Ecuador, French Guiana)	***P. princeps* sp. n.** ♀
–	T2 smooth medially, with scrobiculate lines laterally (Figs 68–73) (Ecuador, Peru)............................	***P. pauper* sp. n.** ♀
8(6)	Basal ⅔ of T1 with at least a large smooth area medially, sometimes entirely smooth (Fig. 85)	**9**
–	Basal ⅔ of T1 longitudinally costate across entire width (Fig. 37)	**10**
9(8)	Posterior margin of T2 straight; 3 or 4 terga visible beyond apex of T2 (Figs 25–30) (Belize, Bolivia, Brazil, Costa Rica, Colombia, Ecuador, French Guiana, Panama, Peru, Trinidad and Tobago, Venezuela)	***P. cudo* sp. n.** ♀
–	Posterior margin of T2 distinctly concave (Fig. 6); 2 or 3 terga visible beyond apex of T2 (Fig. 80) (Brazil, Colombia, Ecuador, Paraguay, Peru)	***P. tonsura* sp. n.** ♀
10(8)	T2, including laterotergite, with widespread sparse pilosity (Figs 1–6) (Brazil).............................. ............................	***P. comata* sp. n.** ♀
–	T2 at most with 1 row of setae posteriorly and sparse setation laterally, elsewhere glabrous (Fig. 66)	**11**
11(10)	Posterior margin of T2 distinctly concave (Figs 61–66)	**12**
–	Posterior margin of T2 straight or only slightly concave (Fig. 20)	**13**
12(11)	T1 costate throughout its length (Fig. 62) (Belize, Bolivia, Brazil, Colombia, Costa Rica, Ecuador, El Salvador, French Guiana, Guatemala, Mexico, Panama, Peru, Trinidad and Tobago, Venezuela)	***P. odo* sp. n.** ♂♀
–	T1 evenly costate across anterior 1/3 to 1/2, smooth posteriorly (Fig. 54) (Bolivia, Costa Rica, Ecuador, Paraguay, Venezuela)	***P. hjalmr* sp. n.** ♀
13(12)	Metasoma with 2 or 3 terga visible beyond apex of T2; frons below median ocellus with two parallel rows of setiferous punctures (Figs 19–24) (Bolivia, Costa Rica, Ecuador, French Guiana)	***P. cranos* sp. n.** ♀
–	Metasoma with 4 or 5 terga visible beyond apex of T2 (Fig. 8); two rows of setiferous punctures below median ocellus not parallel (Fig. 12)	**14**
14(13)	Two rows of setiferous punctures below median ocellus converging ventrally; T1 flat at margin with T2; large species, body length 2.8 to 3.0 mm (Figs 13–18) (Brazil).........................	***P. corys* sp. n.** ♀
–	Two rows of setiferous punctures below median ocellus converging medially and then diverging ventrally; T1 slightly swollen at margin with T2; body length 1.4 to 1.6 mm (Figs 7–12) (Paraguay)	***P. constellata* sp. n.** ♀

## Supplementary Material

XML Treatment for Phanuromyia

XML Treatment for Phanuromyia comata

XML Treatment for Phanuromyia constellata

XML Treatment for Phanuromyia corys

XML Treatment for Phanuromyia cranos

XML Treatment for Phanuromyia cudo

XML Treatment for Phanuromyia dissidens

XML Treatment for Phanuromyia galeata

XML Treatment for Phanuromyia galerita

XML Treatment for Phanuromyia hjalmr

XML Treatment for Phanuromyia krossotos

XML Treatment for Phanuromyia odo

XML Treatment for Phanuromyia pauper

XML Treatment for Phanuromyia princeps

XML Treatment for Phanuromyia tonsura

XML Treatment for Phanuromyia tubulifer

## Appendix 1

**Table:** URI Table matching terms and concepts used in this revision with the Hymenoptera Anatomy Ontology database.

acetabular carina	http://purl.obolibrary.org/obo/HAO_0000292
antenna	http://purl.obolibrary.org/obo/HAO_0000101
area	http://purl.obolibrary.org/obo/HAO_0000146
body	http://purl.obolibrary.org/obo/HAO_0000182
body length	http://purl.obolibrary.org/obo/HAO_0002413
carina	http://purl.obolibrary.org/obo/HAO_0000188
coriaceous	http://purl.obolibrary.org/obo/HAO_0002379
costa	http://purl.obolibrary.org/obo/HAO_0000225
cuticle	http://purl.obolibrary.org/obo/HAO_0000240
depression	http://purl.obolibrary.org/obo/HAO_0000241
egg	http://purl.obolibrary.org/obo/HAO_0000286
eye	http://purl.obolibrary.org/obo/HAO_0000217
frons	http://purl.obolibrary.org/obo/HAO_0001044
frontal depression	http://purl.obolibrary.org/obo/HAO_0000911
head	http://purl.obolibrary.org/obo/HAO_0000397
impression	http://purl.obolibrary.org/obo/HAO_0000417
lamella	http://purl.obolibrary.org/obo/HAO_0000188
laterotergite	http://purl.obolibrary.org/obo/HAO_0000493
mandible	http://purl.obolibrary.org/obo/HAO_0000506
margin	http://purl.obolibrary.org/obo/HAO_0000510
median ocellus	http://purl.obolibrary.org/obo/HAO_0000526
mesepisternum	http://purl.obolibrary.org/obo/HAO_0001872
mesopleural pit	http://purl.obolibrary.org/obo/HAO_0001358
mesoscutellum	http://purl.obolibrary.org/obo/HAO_0000574
mesoscutum	http://purl.obolibrary.org/obo/HAO_0001490
mesosoma	http://purl.obolibrary.org/obo/HAO_0000576
metascutellum	http://purl.obolibrary.org/obo/HAO_0000625
metasoma	http://purl.obolibrary.org/obo/HAO_0000626
metasomal tergite	http://purl.obolibrary.org/obo/HAO_0002005
mouthparts	http://purl.obolibrary.org/obo/HAO_0000639
ocellus	http://purl.obolibrary.org/obo/HAO_0000661
ovipositor	http://purl.obolibrary.org/obo/HAO_0000679
pilosity	http://purl.obolibrary.org/obo/HAO_0001990
pit	http://purl.obolibrary.org/obo/HAO_0000241
propodeum	http://purl.obolibrary.org/obo/HAO_0001248
sculpture	http://purl.obolibrary.org/obo/HAO_0000913
segment	http://purl.obolibrary.org/obo/HAO_0000929
sternaulus	http://purl.obolibrary.org/obo/HAO_0001509
suture	http://purl.obolibrary.org/obo/HAO_0000982
tergite	http://purl.obolibrary.org/obo/HAO_0001783
tooth	http://purl.obolibrary.org/obo/HAO_0001019
wing	http://purl.obolibrary.org/obo/HAO_0001089

## Supplementary materials

Supplementary material 1 Occurrences Data type: DarwinCore Archive Explanation note: Label data for specimens used in study. This dataset is made available under the Open Database License (http://opendatacommons.org/licenses/odbl/1.0/). The Open Database License (ODbL) is a license agreement intended to allow users to freely share, modify, and use this Dataset while maintaining this same freedom for others, provided that the original source and author(s) are credited.Katherine C. Nesheim, Lubomír Masner, Norman F. Johnson
